# Skeletal Class II Malocclusion: From Clinical Treatment Strategies to the Roadmap in Identifying the Genetic Bases of Development in Humans with the Support of the Collaborative Cross Mouse Population

**DOI:** 10.3390/jcm12155148

**Published:** 2023-08-06

**Authors:** Iqbal M. Lone, Osayd Zohud, Kareem Midlej, Peter Proff, Nezar Watted, Fuad A. Iraqi

**Affiliations:** 1 Department of Clinical Microbiology and Immunology, Sackler Faculty of Medicine, Tel Aviv University, Tel Aviv 6997801, Israel; iqbalzoo84@gmail.com (I.M.L.); osaydzohud@gmail.com (O.Z.); kareemmidlej@mail.tau.ac.il (K.M.); 2 Department of Orthodontics, University Hospital of Regensburg, 93053 Regensburg, Germany; peter.proff@klinik.uni-regensburg.de; 3 Center for Dentistry Research and Aesthetics, Jatt 4491800, Israel; nezar.watted@gmx.net; 4 Department of Orthodontics, Faculty of Dentistry, Arab America University, Jenin 34567, Palestine; 5 Gathering for Prosperity Initiative, Jatt 4491800, Israel

**Keywords:** skeletal class II malocclusion, etiology, treatment, quantitative trait loci, collaborative cross mouse model

## Abstract

Depending on how severe it is, malocclusion, which may involve misaligned teeth, jaws, or a combination of the two, can hurt a person’s overall facial aesthetics. The maxillary molar develops before the mandibular molar in class II malocclusion, which affects 15% of the population in the United States. With a retrusive mandible, patients typically have a convex profile. The goal of this study is to classify the skeletal and dental variability present in class II malocclusion, to reduce heterogeneity, present the current clinical treatment strategies, to summarize the previously published findings of genetic analysis, discuss these findings and their constraints, and finally, propose a comprehensive roadmap to facilitate investigations aimed at determining the genetic bases of malocclusion development using a variety of genomic approaches. To further comprehend the hereditary components involved in the onset and progression of class II malocclusion, a novel animal model for class II malocclusion should be developed while considering the variety of the human population. To overcome the constraints of the previous studies, here, we propose to conduct novel research on humans with the support of mouse models to produce contentious findings. We believe that carrying out a genome-wide association study (GWAS) on a large human cohort to search for significant genes and their modifiers; an epigenetics-wide association study (EWAS); RNA-seq analysis; integrating GWAS and the expression of quantitative trait loci (eQTL); and the testing of microRNAs, small RNAs, and long noncoding RNAs in tissues related to the skeletal class II malocclusion (SCIIMO) phenotype, such as mandibular bone, gum, and jaw in humans and the collaborative cross (CC) mouse model, will identify novel genes and genetic factors affecting this phenotype. We anticipate discovering novel genetic elements to advance our knowledge of how this malocclusion phenotype develops and open the venue for the early identification of patients carrying the susceptible genetic factors so that we can offer early prevention treatment strategies.

## 1. Introduction

Following dental cavities and periodontal disorders, malocclusion is the third-most prevalent oral health issue [1]. Skeletal class II malocclusions (SCIIMO) account for over one-third of all malocclusions observed globally and are more common in Caucasians than in other races [2]. Accordingly, in general dentistry practice, class II malocclusion patients make up about one-third of patients needing orthodontic treatment [3]. This form of malocclusion is caused by a variety of causes. Still, most research reports have linked it to mandibular deficiency (mandibular retrognathia is the leading cause, rather than maxillary prognathism), necessitating the adoption of mandibular advancement appliances [4,5,6], which significantly impairs patients’ ability to chew food effectively. Development modification, which involves suppressing maxillary growth and/or stimulating mandibular growth, can treat skeletal class II malocclusion in the preadolescent stage [4,6,7]. Temporomandibular joint (TMJ) diseases are thought to be predisposed by class II malocclusion. The jaw joints, surrounding muscles, and ligaments all experience pain and tenderness when you have TMJ dysfunction (TMD). Teeth grinding, jaw injuries, arthritis, and normal wear and tear are some of the causes. TMJ treatment differs from patient to patient and may entail prescription medicine, physical therapy, creating a unique mouth guard, or even jaw surgery.

Extraoral headgear, removable appliances, and fixed functional appliances (FFAs) are a few examples of orthopedic appliances that can be used for this [8]. There is conflicting evidence about the therapeutic efficacy of removable functional appliances, and they are significant and very unpleasant to kids. Positive treatment effects on mandibular growth, including effective condylar growth [9,10,11] and enhanced mandibular length [12,13,14], have been observed in several investigations. Other researchers, however, could not detect clinically significant effects [15,16,17,18]. A similar debate exists concerning the impact of this effect, according to specific investigations of appliances on the maxillary jaw. There is a limit [19,20], although other studies disagree [21]. There is general agreement that these appliances cause the mandibular incisors to procline and the maxillary incisors to recline [22]. The exact opposite is true concerning their dentoalveolar effects, similar to how FFAs affect the dentoalveolar system more than the skeletal system [23,24,25]. Overall, the evidence from systematic reviews and meta-analyses supports that neither FFAs nor detachable equipment generate pure skeletal alterations; rather, their effects are essentially dentoalveolar [20,22,26,27,28]. The shortcomings of traditional orthopedic and orthodontic mechanics have been removed with the creation and use of skeletal anchorage devices in orthodontics. To counteract the effects of FFAs on the mandibular incisors, skeletal anchoring devices were utilized on a single jaw [29,30,31,32]. They have recently been applied to both jaws with the help of intermaxillary protracting force to enhance the skeletal effects of the intended orthopedic treatment [7,33,34,35]. The biomechanical purpose of using skeletal anchorage is to transfer the applied force to the underlying bone, either to prevent the unwanted effects of a direct force applied to the fixed functional appliances or to directly transfer the force to the jawbone to produce the necessary growth modification. A few systematic studies and meta-analyses have evaluated the effects of employing skeletal anchorage on a single jaw to support the mandibular advancement appliances on the skeletal and dentoalveolar systems [36,37,38]. Mini screws or miniplates can now be attached to both jaws using novel techniques, with the ultimate goal of producing purely skeletal effects. The evidence regarding the skeletal and dentoalveolar effects of employing skeletal anchorage for increasing the skeletal effect by applying the stresses directly or indirectly to the underlying bone of both jaws, however, has not yet been examined by a single systematic review.

**Figure 1:**
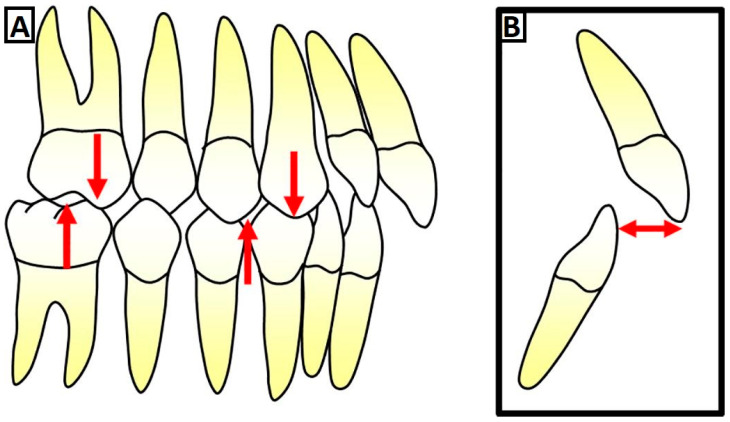
Definition of a class II dysgnathy. In class II molar relationship, the mesiobuccal cusp of the maxillary first permanent molar occludes mesial to the buccal groove of the mandibular first molar (as shown by red arrows) (**A**), which, in general, results in observing an overjet (red arrow shows the space between the front teeth) (**B**).

**Figure 2:**
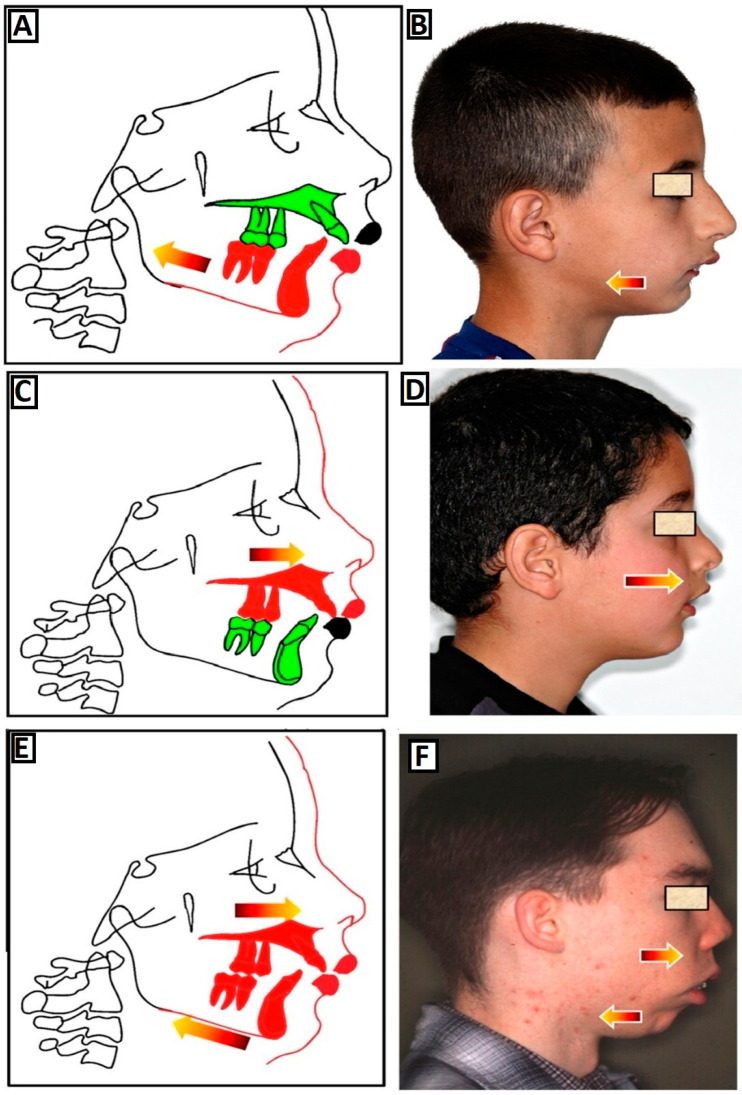
The causes of class II dysgnathy. (**A**,**B**) The class II skeletal malocclusion phenotype is characterized by mandibular retrusion (mandibular retrognathism), with (**A**) a diagram and (**B**) a lateral view of a patient with this phenotype. (**C**,**D**) The class II skeletal malocclusion phenotype is characterized by a maxillary protrusion (maxillary prognathism), with (**C**) a diagram and (**D**) a lateral view of a patient with this phenotype. (**E**,**F**) The class II skeletal malocclusion phenotype is characterized by a combination of maxillary protrusion (maxillary prognathism) and mandibular retrotrusion (mandibular retrognathism), with (**E**) a diagram and (**F**) a lateral view of a patient with this phenotype. The arrows show if there is a mandibular retrognathism or/and maxillary prognathism.

The skeletal class II malocclusion (SCIIMO) phenotype (Figure 1) is heterogeneous and is usually characterized by mandibula retrusion (mandibular retrognathism) (Figure 2A,B), maxillary protrusion (maxillary prognathism) (Figure 2C,D), or a combination thereof (Figure 2E,F) and may present as an isolated feature or as part of a syndrome to reduce the heterogeneity and facilitate investigations aiming at determining the cause of malocclusion. SCIIMO diagnosis and treatment planning may use advanced technologies in order to predict tooth and bone modifications [39]. The goal of this research is to classify the skeletal and dental diversity found in class II malocclusion to separate phenotypes; present the clinical treatment strategies of these complex phenotypes; identify the current published data on the genetics of SCIIMO, its findings, and constraints; and finally, suggest a comprehensive approach for identifying the genetic components involved in class II malocclusion development in humans with the support of the suggested mouse model.

## 2. Clinical Treatment Strategies

According to the patient’s age, compliance, underlying skeletal pattern, and dental traits, the treatment of a SCIIMO should address the etiology. It is essential to consider the aesthetics of smiling. The primary dental goals of therapy include stable repair of the overjet, overbite, and occlusion.

### 2.1. Treatments Options for Skeletal Class II Malocclusion

Based on our knowledge in the field of orthodontics, in principle, there are several therapeutic approaches for the treatment of SCIIMO dysgnathy, which are described here:

I. A causal therapeutic approach in the sense of a targeted influence on the growth component. For this purpose, functional orthodontic devices for ventral development of the mandible with simultaneous growth inhibition of the upper jaw in the sagittal and vertical directions (HG effect) are used in the therapy of class II dysgnathy.
II. A dentoalveolar therapeutic approach in the sense of conservative space creation measures. The focus here is on the distalization of the 6-year molars up to the neutral position so that, after subsequent distalization of the support zone and retraction of the front of the upper jaw, a correction of the sagittal front tooth step is possible. Many factors, such as space conditions, must be considered for this treatment measure; the sagittal and vertical cranial structures, as well as dentofacial aesthetics, are considered.
III. A dentoalveolar therapy approach in the sense of absolute space creation measures by extracting permanent teeth. In addition to the systematic extraction of the premolars, the extraction of two premolars should only be considered in the upper jaw if the initial situation is appropriate; the goal of treatment concerning the occlusion is therefore neutral on the canines and 1PB distal on the first malar. For these treatment measures, as with the previously described options, many factors must also be considered.
IV. A skeletal correction in the sense of jaw surgery compensation. If the growth for the correction of a pronounced class II is no longer available and a conservative or an absolute space creation measure is not indicated, the treatment of the dysgnathia is only possible in this case with a combined orthodontic and maxillofacial surgical treatment.

**Figure 3:**
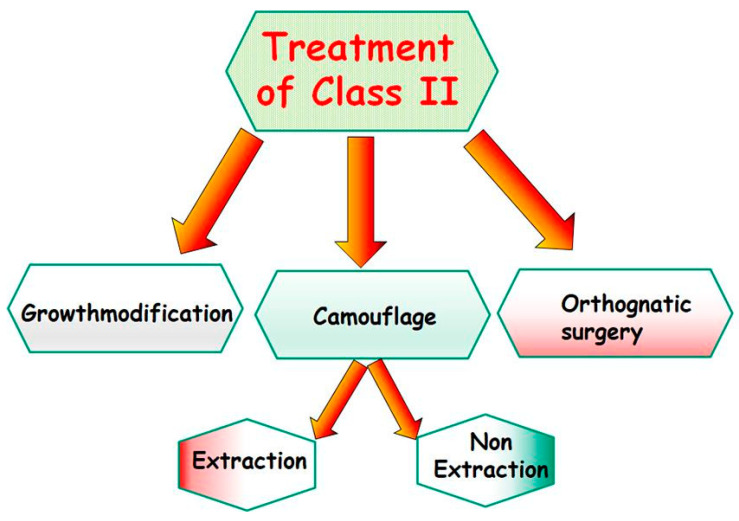
Treatment options and strategies of class II dysgnathy. This depends on age (growth), cause, compliance, and treatment goals.

**Figure 4:**
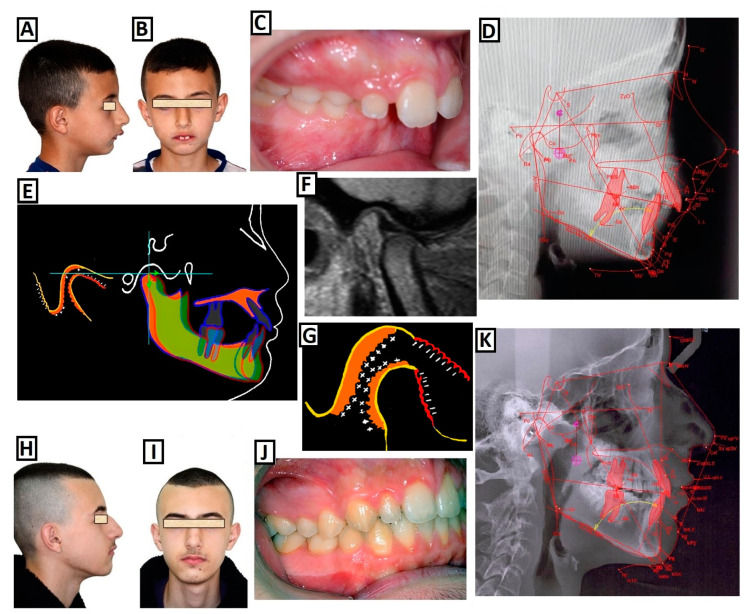
Treatment of class II dysgnathy during the growing ages through growth-influencing methods. (**A**–**D**) Before treatment: (**A**) lateral view photo of the patient, (**B**) frontal view photo of the patient, (**C**) intraoral photo, and (**D**) cephalometric radiograph. (**E**–**G**) The use of the device to influence growth: creating new conditions for the jaw condyles and, thus, intensifying growth in the fossa and condyles. Mandibular advancement through the construction bite causes bone resorption and bone apposition in the fossa and condyles. The MRI image shows the new position of the condyles with the construction bite. (**H**–**K**) After treatment: (**H**) lateral view photo of the patient, (**I**) frontal view photo, (**J**) intraoral photo, and (**K**) cephalometric radiograph.

**Figure 5:**
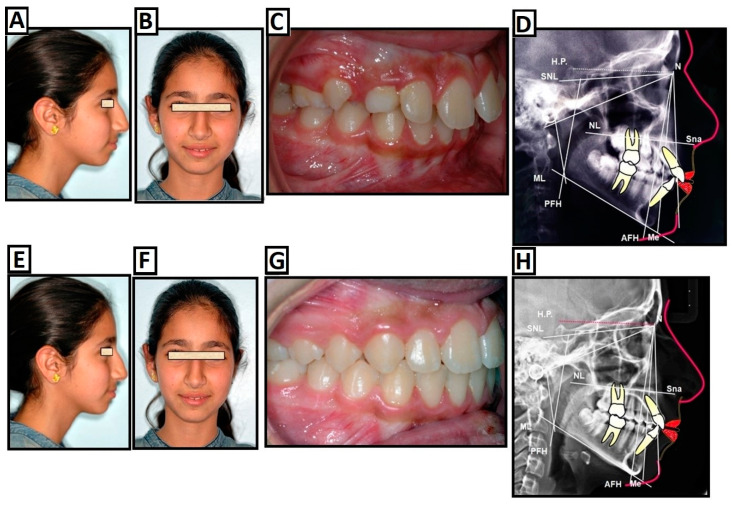
(**A**–**F**) Camouflage therapy of a class II dysgnathy, non-extraction, by destabilizing the teeth in the upper jaw. (**A**–**D**) Before treatment: (**A**) lateral photo of the patient, (**B**) frontal photo of the patient, (**C**) intraoral photo, and (**D**) cephalometric radiograph. (**E**–**H**) After treatment: (**E**) lateral photo of the patient, (**F**) frontal photo of the patient, (**G**) intraoral photo, and (**H**) cephalometric radiograph.

**Figure 6:**
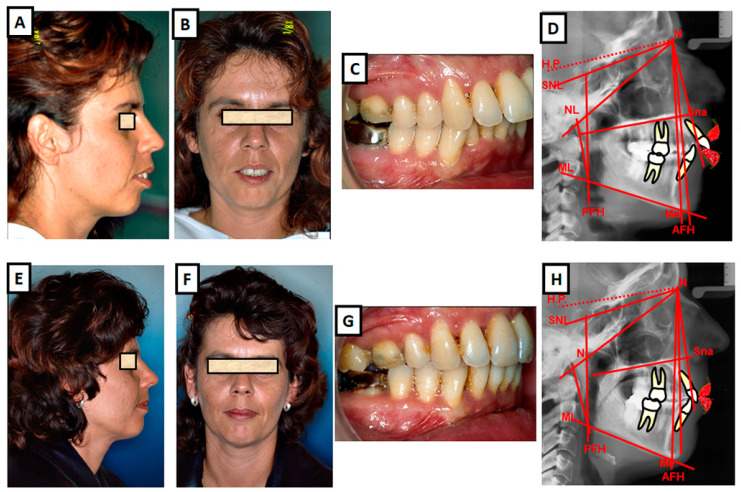
Camouflage therapy of class II dysgnathy by extracting permanent teeth in the upper jaw. (**A**–**D**) Pretreatment images: (**A**) lateral view of a 45-year-old patient with a convex profile, (**B**) frontal view showing poor lip closure due to an anterior maxilla labial tilt and increased overjet, (**C**) class II occlusion, and (**D**) cephalometric image revealing a disharmonious skeletal arrangement. (**E**–**H**) Posttreatment images: (**E**–**G**) relaxed supramental and competent lip closure, and (**H**) occlusion at the end of treatment exhibiting a stable class I occlusion with a physiological overjet.

**Figure 7:**
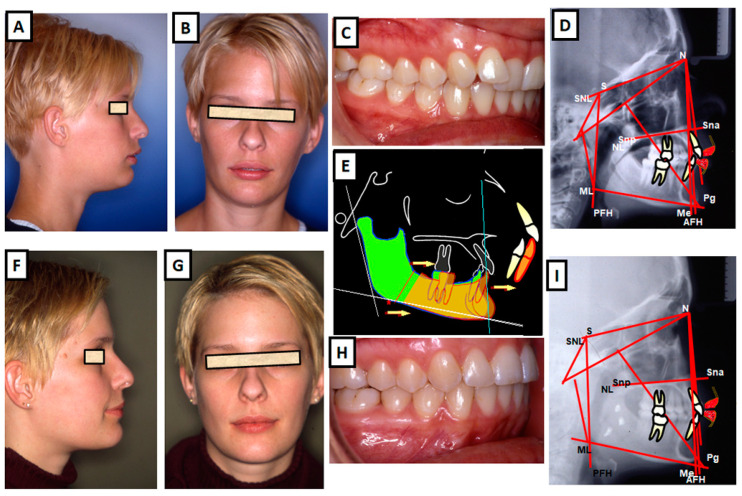
Combination therapy (orthodontics and surgery) of class II dysgnathy. (**A**–**D**) Pretreatment images: (**A**) lateral view of a 21-year-old patient with class II dysgnathy, (**B**) frontal view demonstrating poor lip closure and increased overjet, (**C**) class II occlusion, and (**D**) cephalometric analysis revealing a disharmonious skeletal relationship. (**E**–**I**) Posttreatment images: (**E**) lateral view showing an improved profile with harmonious soft tissue and hard tissue structures, (**F**) frontal view demonstrating enhanced lip closure and reduced overjet, (**G**) class I occlusion, (**H**) cephalometric analysis indicating an improved skeletal harmony, and (**I**) close-up view highlighting the corrected sagittal relationship without compromising the vertical ratio.

To our knowledge, there are several treatment strategies, and the type of treatment depends on several parameters: function, growth, facial aesthetics, and compliance. Currently, the main treatment options/procedures for correcting a SCIIMO include (I) growth modification, (II) orthodontic camouflage therapy, and (III) surgical orthodontics, as shown in Figure 3. Treatment strategies for class II dysgnathy, in children include through growth-influencing approaches (Figure 4), camouflage treatment for class II dysgnathy by non-extracting permanent teeth (Figure 5), camouflage therapy of a class II dysgnathy by extracting permanent teeth (Figure 6), and combination therapy (orthodontics and surgery) of class II dysgnathy (Figure 7).

### 2.2. Correction of Increased Interincisal Angle

According to orthodontics knowledge and basic agreement, the lower incisal margins should be within 2 mm of the maxillary central incisor centroid, and the interincisal angle should be around 135° to produce an occlusal stop effectively. Proclination of the maxillary and mandibular anterior segments and enhanced palatal root torque on the maxillary incisors are methods to accomplish this. To torque the incisor roots palatally, sufficient palatal cortical bone is necessary, which can place a strain on the anchoring needs. Due to the possibility of losing periodontal attachment, patients with a thin gingival biotype should refrain from performing large labial motions in the mandibular labial segment. The inter-canine breadth is unstable with any significant increase; therefore, long-term permanent retention will be needed.

### 2.3. Correction of Increased Overbite

The standard methods for treating deep dental bites include incisor intrusion and molar extrusion. In practice, multiple strategies are used, because achieving absolute incursion is challenging. The patient’s incisor displays while smiling and at rest should be considered when deciding whether to extrude posteriorly or intrude anteriorly. Mandibular incisor proclination can lessen an overbite, but caution must be exercised to ensure that the proper interincisal angle is achieved. Patients with little potential for growth typically receive absolute intrusion. Additional arches may be used, such as the Mulligan bypass arch and the Burstone intrusion arch. Clear aligners and fixed appliances can be used with skeletal anchorage tools like mini screws to improve the mechanics of the incursion. As the incisors are kept in place during vertical facial development, molar eruption and aided molar extrusion occur in developing youngsters, and relative intrusion of the anterior section occurs. Using anterior bite turbos or an upper detachable appliance with an anterior bite plane—the latter of which depends on compliance—can also cause guided molar eruption. Molar and premolar extrusion are less stable in non-growing patients, because the interocclusal forces tend to push the molars back in after active appliances are removed [40,41]. This propensity for relapse is lessened, though, if efficient interdigitation, occlusal stops, and restoration of the optimum interincisal angle are accomplished. Molar extrusion may cause the mandible to rotate downward and backward, increasing the height of the lower anterior face. In dolichofacial patients with mild-to-severe mandibular retrognathia, this may be desired, because many class II individuals have brachyfacial features. Compared to prepuberty, puberty is the best time to repair a deep bite [42]. Headgear treatment may be helpful in growing patients with a SCIIMO basis to distalize or hold back the maxillary teeth as the patient grows. Similar to this, a handy device would be helpful to advance the lower arch. The upper incisors must first be proclined to give enough overjet to allow mandibular progress in class II patients before functional appliances can be employed.

The finest removable functional appliances for achieving upper incisor proclination are those with sectional fixed appliances or those with an anterior screw or palatal springs to the maxillary anterior segment. To create a good, stable occlusion and proper tooth alignment after functional treatment, fixed appliances or clear sequential aligner therapy is necessary. As an alternative to fully permanent appliances, fixed class II correctors or class II elastics are a form of treatment. There is a larger tendency for these patients to be treated by non-extraction, because those with a class II malocclusion frequently have less crowding and straight to concave lower-third facial features. More posteriorly positioned teeth are typically selected when extractions are necessary. Patients who are not growing and have a substantial SCIIMO base and a severe dental discrepancy may need orthognathic surgery. According to preliminary studies on rats, injections of botulinum toxin into the masseter muscles reduced the occlusal forces and caused the molars and incisors to emerge supra-normally [43]. To assess the impact and stability of botulinum toxin-induced dental extrusion, more human subject-based research is necessary. A severe anterior overbite, inter-canine width collapse, and the return of anterior crowding must be avoided long after therapy. If molar extrusion is the primary treatment goal, a removable retainer with an anterior bite plane might be helpful to lessen overbite relapse.

## 3. Etiology of SCIIMO Development

The emergence of SCIIMO has a significant hereditary component. Twin studies have shown that this malocclusion is inherited either as a variable expressive autosomal-dominant trait or as a polygenetic expression of critical morphological features [44,45]. Additionally, specific characteristics of the musculoskeletal system, such as the size and form of the jaw, and the development of the teeth, like their size, shape, and number, are genetically predetermined. This malocclusion may have developed as a result of several dentoskeletal causes. The orofacial forces’ equilibrium can be upset by epigenetic factors, such as the presence of a high lip line, an expressive lower lip that resembles a strap, and hyperactivity of the mentalis muscles, which can cause the maxillary incisors to retrocline [44,45]. This could lead to a more significant overbite and increase the interincisal angle. The maxillary central incisors are typically retroclined in SCIIMO, while the lateral incisors next to them are typically proclined or angled averagely. However, these teeth can also become retroclined if the lower lip line completely encloses the incisal part of the maxillary lateral incisors. Class II malocclusions, in contrast to other malocclusions, are more frequently linked to congenital tooth anomalies like hypodontia, microdontia, deformities of the maxillary lateral incisors, and tooth transpositions [46].

Additionally, underlying class II malocclusions are present in 44% of people with palatally displaced permanent canines. This may be partially brought on by the enlarged maxillary transverse dimensions and/or lateral incisor microdontia [47]. Class II malocclusion was compared to a syndrome by [44,45] due to the prevalence of repeated morphometric characteristics in those with this malocclusion. It should be emphasized that not every person with a class II malocclusion will display all of the mentioned characteristics and that many people’s malocclusions fall into more than one of Angle’s four categories.

### 3.1. Genetics and Heritability of Class II Malocclusion

Heritability is the percentage of a trait attributed to the heredity of a given phenotype/trait [44]. When assessing the impacts of genetic and environmental interactions and the effects of environmental factors alone on particular traits, twin studies—particularly those of monozygotic twins—are significant and helpful. Monozygotic twins’ differences in their morphological structures, which have identical genetic compositions, would be caused by the environment, as opposed to dizygotic twins’ differences, which have environmental and genetic influences [48]. To ascertain the degree to which the craniofacial complex is inherited, twin studies and progenitors–progeny familial heritability investigations that demonstrate the impact of cumulative genetic factors by studying the differences or divergences observed between parents and their children are also useful [49]. The degree to which the development of class II malocclusion is influenced by genetics as opposed to environmental factors remains uncertain [50].

Monozygotic twins showed good concordance in malocclusion features, according to studies on twins with class II conditions, but dizygotic twins showed 90% discordance in these conditions [45]. In twin studies, research on bony contours, which identified regions of bone deposition, resorption, and potential growth sites, was conducted to identify structures with the strongest hereditary influence [48]. The lingual midline, lateral aspect of the mandibular ramus, and anterior contour of the mandibular bone were delineated areas in the twin studies by Watnick that were demonstrated to be more under the genetic influence than others, like the antegonial notch [45]. The number of third molars present, the size of the tooth crown, the width of the arch, and the malformation of the maxillary lateral incisor have all demonstrated substantial heritability (more than 70%) using 17 dental characteristics [51]. Numerous studies have revealed significant familial segregation for class II division, which is possibly caused by an incompletely penetrant autosomal-dominant inheritance (i.e., the percentage of patients in the community that have the disease-causing mutation and display the specified phenotype). But a polygenic inheritance, in which multiple genes work additively, is also a recognized mechanism of heredity [52]. Japanese patients in skeletal classes II and III malocclusions and their parents were investigated by Nakasima et al. in 1982 for lateral and frontal cephalometric characteristics [53]. The skeletal measurements had higher correlation coefficients than the dental measurements. In the class II group, all skeletal parameters in a father and child were significantly associated (the highest correlation value discovered was 0.502), which was consistent with the polygenic method of inheritance [53].

### 3.2. Importance of Genetic Studies

A straightforward Mendelian inheritance pattern can be used to describe and explain discrete features like cystic fibrosis [54]. The continuous and multivariate (polygenic) nature of increasingly complex traits like craniofacial abnormalities leads to a more complicated mode of inheritance [44]. A linkage analysis, a method used in familial studies, compares the genomic areas of affected members of a pedigree to identify the chromosomal regions responsible for a specific genetic characteristic. However, an association analysis, which compares genomic areas in unrelated people with comparable characteristics, can also be used to achieve this goal [55,56].

Single-nucleotide polymorphisms (SNPs), in which a mutation manifests as a single base substitution and generates a single-nucleotide variation in two DNA strands, are the most often observed genetic causes of disease susceptibility [57]. Genomic association studies, which involve linking SNPs with a particularly complicated characteristic, have produced significant advancements in figuring out the genetic basis of numerous complex traits [58]. Pleiotropic refers to the property of a gene to explain numerous phenotypes. Pleiotropy exists in 17% of genes and 5% of SNPs in complex phenotypes [59]. Due to the pleiotropic effect, a harmful gene mutation might induce many morphologic defects [44], complicating the identification of causative mutations that could account for malocclusion. There have been a very small number of genetic investigations on humans to pinpoint the genes that increase malocclusion susceptibility. Compared to class II malocclusion, class III malocclusion has undergone more research to determine the hereditary origins.

A genome-wide linkage investigation was conducted on Korean and Japanese families exhibiting mandibular prognathism. These studies mapped three chromosomal loci located on 1p36, 6q25, and 19p13, which were associated with this phenotype. The highest linkage result came from locus 1p36, which indicated that it was the most likely genomic site for the genes causing mandibular prognathism [60,61]. A subsequent study of these results discovered that mandibular prognathism is linked to the EPB41 gene [61].

Although the genetic causes of mandibular deficits in animals have been shown in other studies, a connection to humans has not yet been shown. To identify the genes responsible for particular symptoms, quantitative trait loci (QTL) investigations have been helpful [62]. For instance, a research design was performed on mice by measuring the mandibular length, utilizing the gonion to menton as landmarks, and discovering substantial genetic links (two significant QTLs) to regions on chromosomes ten and eleven, which explain the variations in size. In addition, more advanced techniques for characterizing the mouse mandible have been carried out using geometric morphometric approaches, which make use of linear and angular measurements, as well as the detection of particular markers, to ascertain what effect they have on the dimensions and form. Using Procrustes superimposition and five morphological landmarks, this multivariate approach was performed to identify the association of QTLs and their influence on the dimensions and form of mouse mandibles. As some chromosomal areas in mice match specific sites in human chromosomes, this knowledge can support ongoing research into how genes regulate the size of human mandibles [63].

A recent study has shown that all affected people in Colombian families with SCIIMO propensity were discovered to be homozygous for an unusual variant, rs1348322, in the *Nog* gene. However, the polymorphism’s precise impact is unknown [64]. Mandibular deficiency’s genetic origin has been researched, as well as the possibility of genetically modifying this preexisting illness in animal models. Mandibular hypoplasia is a common symptom of class II malocclusion; hence, gene therapy is an additional treatment option to encourage condylar growth. Rat condyle growth was promoted and bone formation was encouraged by injecting recombinant adeno-associated virus-mediated vascular endothelial growth factor (rAAV-VEGF). Both the condyle and the mandibular length increased as a result. Gene therapy could help cause higher mandibular growth by either promoting growth or serving as an alternative supply to faulty genes [65]. It is crucial to identify the cause of malocclusion, because doing so will open up more therapy options that can help with malocclusion prevention. Lately, genome-wide association analyses (GWAS) have been employed to identify the particular genetic loci responsible for various oral traits and aberrations [66]. Four loci were found to be linked to the eruption of permanent teeth in children by two distinct GWAS investigations, and five loci were found to be significant in the eruption of primary teeth. There were two loci that both groups shared [67,68]. A greater understanding of the genetic involvement in the emergence of specific malocclusion phenotypes will contribute to determining the origin of malocclusion and aid in its prevention [44]. To reduce the heterogeneity, prevent the incorrect classification of those who are affected, and facilitate genetic and environmental studies, these malocclusion phenotypes must be appropriately described. Prospective investigations into the genetic and environmental factors contributing to malocclusion can leverage the current technological advancements, allowing for the study of well-defined phenotypes. These advancements enable the comprehensive assessment of entire genomes through the high-throughput genotyping of SNPs or genome sequencing, facilitating the examination of human genetic diversity. This will shed light on the pathogenesis and etiology of malocclusion [69,70,71].

## 4. A Mouse Model for Studying Class II Malocclusion

The accumulated influence and intricate interplay of many genes and environmental variables make it difficult to conduct controlled and standardized genetic studies of complex human disorders. The precise genes involved in the bulk of complex illness vulnerability and the mechanism translating genetic influences into vulnerability to diseases are largely unclear, despite significant discoveries of genetic risk factors for some diseases. A helpful tool for analyzing the genetic underpinnings of complex traits and diseases is the mouse model (multifactorial phenotypes). Using comparative and ortholog analyses, it is possible to translate a set of mouse genes related to and underlining a particular phenotype in humans.

The mandible size in mice is regulated by genes discovered on chromosomes ten and eleven, which correspond to Homo sapiens chromosomal regions 12q21 and 2p13, accordingly, according to a recent study by Dohmoto et al. [63]. Unfortunately, to our knowledge, no study has taken this information further and tried to search for ortholog genes in human cohorts and find the underlying genes for these phenotypes. Orthodontists would be better able to choose treatment options for SCIIMO if they could clinically determine whether a patient had strong risk factors for mandibular overgrowth. Despite the patient needing the long-term use of appliances, orthodontists may decide to perform orthodontic surgery on a developing patient with a SCIIMO if the patient possesses hereditary variables, such as variations in candidate genes. To repeat and carry out these analyses by gender, age group, and ethnicity, we now intend to keep working to increase the examined population (cohort). We suggest that, by incorporating a sizable subject population comprising a thoroughly characterized, multiethnic sample, encompassing individuals of European, Middle Eastern, Hispanic/Latino, mixed African, and Asian ancestries, the analysis will be strengthened, and there will be a better chance of identifying the genetic factors that contribute to the development of skeletal class II malocclusion in humans.

### 4.1. QTL Analysis in a Mouse Model

According to earlier studies, the mandible should exhibit spatially structured effects on genes [72]. Signaling interactions control how the immature mesenchyme buds that make up the face’s rudiments eventually grow into the complex array of bone and cartilage structures that, together with muscle and additional tissues, form the human face. Since polygenes have a role in facial development, including mandibular growth, it is challenging to determine the association of phenotypes with the genes governing the mandibular form. With the quantitative effects depending on polygenes like body weight, alcoholism susceptibility, etc., QTL analysis has proven particularly successful in discovering chromosomal areas [62]. Mice that are recombinantly inbred (RI) help examine complicated variables like body weight. The controlled mating of a stochastic selection of mating F2 progeny pairings of a hybrid formed by two various strains of mice with high homozygosity yields RI strains.

When measuring the separation across the points representing the menton and the gonion, Dohmoto et al. (2002) concentrated on determining the areas of the genome responsible for controlling the mandibular anterior–posterior size [63]. According to the presented results from the study of the genetics of mandibular morphology (the distance between the menton and gonion) utilizing QTL with the SMXA RI strain, both males and females had two significant QTL and a potential QTL located on chromosome 10. Two substantial QTL were discovered at the distal area of the eleventh chromosome in the females. These QTLs could be useful for researching class II malocclusion and creating preventative measures.

Table 1 summarizes, to our knowledge, the currently mapped QTL; their positions; and the estimated ranges of significance for the dimensions (mean of the two sides) of the mandible characters (M), shape (SH), centroid size (C), and facial shape principal components (PCs) given as map distances from the nearest proximal marker and from the centromere after a QTL analysis on mice.

### 4.2. The Collaborative Cross Mouse Cohort Represents a Valuable Resource for Conducting a System Genetic Study on Class II Malocclusion

Mice have shown similar vulnerabilities to numerous infections and environmental factors to humans; therefore, many restrictions in the studies of human populations can be overcome. Using comparative and ortholog analyses, the genes linked to and underlining a particular feature in mice can be found in humans. Standard laboratory mouse lines, however, contain little genetic variety and are, therefore, only marginally relevant for researching diverse genetic manifestations within complex disorders. To address this, collaborative cross (CC), unique, very genetically varied recombinant inbred mouse lines were created. The CC mouse lines were developed as an emerging technique for precise genomic mapping and characterization of the genetic components behind complex phenotypes, focusing on those of critical importance to human health. The requirement to simulate genetic diversity led to the formation of the mouse CC genetic reference population (GRP). This GRP source is a large panel of recombinant inbred (RI) strains created particularly for complex trait research from a genetically heterogeneous group of eight founder breeds [75,76,77], suggesting a strength over any previously reported approach [78].

This unique resource is a large panel of recombinant inbred (RI) strains derived from a genetically diverse set of eight founder strains and designed specifically for complex trait analyses [78,79] and suggests a greater power than any reported approaches earlier [80]. The founder strains are genetically varied, comprising three wild, generated strain founders (CAST/Ei, PWK/PhJ, and WSB/EiJ) and five common laboratory strains (A/J, C57BL/6J, 129S1/SvImJ, NOD/LtJ, and NZO/HiLtJ). The substantial genetic variation in the final group of CC mice is a result of this divergence.

**Figure 8:**
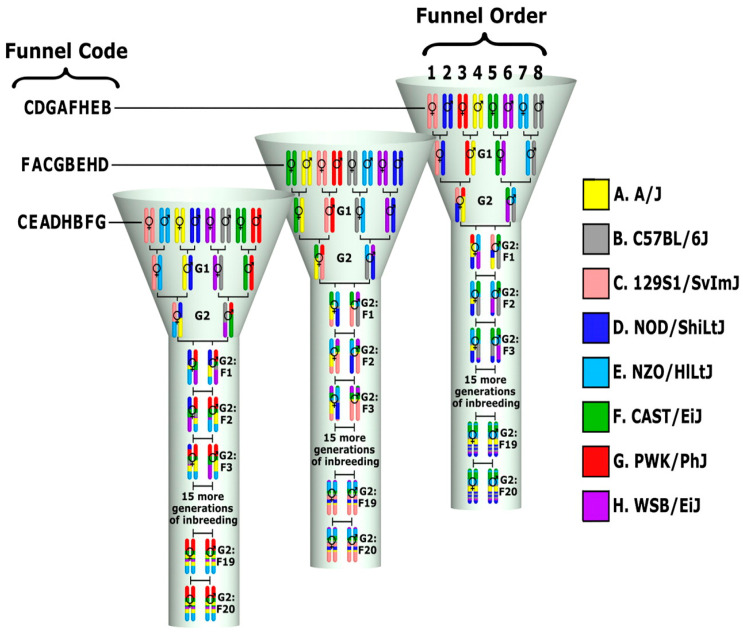
A unique design breeding funnel scheme showing the development of the collaborative cross (CC) mouse model. This breeding approach is designed to randomize the genetic makeup of each inbred line. A single breeding funnel results in one CC recombinant inbred line that represents the genomes of the eight CC mice founders. The eight founder strains are arranged in different positions (1–8) in each line, i.e., their order is randomized and not repeated across lines, and this order determines the funnel code based on a single letter code for each line. In a funnel breeding scheme, the genetic contributions of all eight founder strains are incorporated after the G2 generation. A recombinant inbred line is created following 20 generations of inbreeding [79].

**Figure 9:**
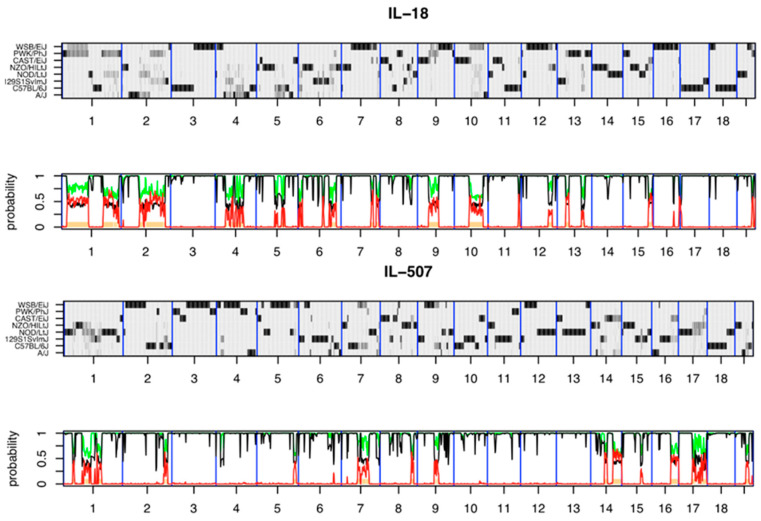
Reconstructions of the genomes of the representative CC lines IL-18 and IL-507 from the hidden Markov model (HMM) were implemented by HAPPY. The *x*-axis shows the 19 autosomes. Each reconstruction is represented by two panels: the top panel *y*-axis shows the 8 CC founders, and the probability of descent from a founder at a locus is represented by the shade of grey, with white = 0 and black = 1. The regions where a single haplotype predominates appear as dark horizontal bands; loci with residual heterozygosity or where the founder haplotypes are indistinguishable are paler grey. The lower panel indicates local heterozygosity (red), the posterior probability of the most probable founder (black), and the sum of the most probable pair of founders (green) [80].

Figure 8 shows how a carefully thought-out breeding plan resulted from introducing the genomes of eight CC founder strains into a single CC line. It is expected to attain nearly 99% homozygosity after more than 20 generations of brother–sister mating after the introduction of the parental founder genes during the G2-F1 stage, with a fairly equal contribution from the eight founder lines [80,81] (Figure 9). An entirely different genetic mosaic can be created in a new CC line by altering the sequence of the founder strains during the outbreed mating stage. As a result, each CC line’s genetic component is distinct and has genotypes that are stable and well known. Compared to previous mouse sets, this genetic reference population (GRP) contains a comparatively high degree of recombination events (4.4 million SNPs are segregated between the founders), two times the number of genetic differences present in the average human population (about 36 million SNPs) [77,78]. The latest QTL assessment stimulation research utilizing the CC population revealed that the mapped interval’s resolution may be less than 1 MB [80,81,82,83,84,85].

The CC population has had roughly fourfold improved mapping, which enhances the precision when locating QTL on the genetic map. Due to the population’s inbreeding origins, every genetic characteristic is homozygous, which amplifies the genetic variation for each QTL. Additionally, it is feasible to lessen the influence of environmental sources of variance by phenotyping a more significant number of individuals within each line. Using recombinant inbred lines (RILs) considerably increases the efficacy of the mapping power compared to standard F2 mapping populations.

It should be possible to run GWAS on CC breeds, identify crucial quantitative trait loci (QTL), discover candidate genes, and define modifiers for the key genes linked to these SCIIMO features while under minimal levels of external sources of variance. It is strongly believed that the tremendous genetic diversity of the CC mice strains offers a good foundation for finding novel genetic loci connected to these described traits and going forward with the confirmation by utilizing conditional knockout techniques and mouse knockout genes.

### 4.3. Future Direction of Developing a Novel Model for Mapping Significant and Modifier Genes Associated with Skeletal Class II Malocclusion Using the CC Model

**Figure 10:**
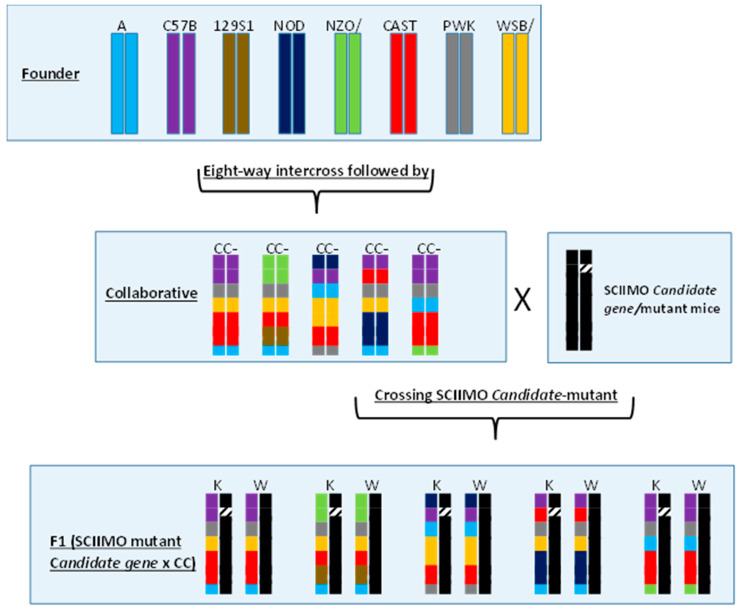
Breeding scheme for the generation of F1 (SCIIMO mutant gene^+/−^ x CC). Different colors represent the genotypes of the respective chromosomes of the eight founder strains. The generated CC lines (assigned *CC1, CC2, CC3, CC4,* etc.) are crossed with *Smad*4 mutant inbred mice to generate an F1 (SCIIMO mutant gene^+/−^ x CC) and a control F1 (SCIIMO mutant gene^+/+^ x CC) population. Since each F1 (SCIIMO mutant gene x CC) mouse has one chromosome from SCIIMO mutant gene mutant inbred mice and the second chromosome from individual CC lines, any phenotypic and genotypic differences between the F1 crosses from the different CC lines will be due to the CC genotypes alone. KO: knockout, WT: wild-type, and CC: collaborative cross.

A potential method for comprehending the multiplicity of biological data that support complex features in genetically divided populations is system genetics. This method employs a variety of experimental and statistical approaches to precisely measure phenotypes in these genetically segregated populations that are anticipated to vary for variables of interest such as the transcript, protein, or metabolite quantities. The first thorough understanding of the molecular architecture of complex characteristics has been made possible by system genetics investigations. They are useful for finding the genes, pathways, and networks that are at the root of widespread disorders. Here, we propose to use the power of the CC lines for mapping genes associated with skeletal class II malocclusions (SCIIMO) with two separate approaches:

1. We propose to perform a traditional search of candidate genes associated with SCIIMO using GWAS, as conducted successfully in previous publications [75,76,77,78,79,80,81,82,83,84].
2. Crossing a mouse carrying mutant genes known to be associated with SCIIMO (e.g., EPB41, Nog, or any other genes listed in Table 1) with a set of mice possessing diverse genetic backgrounds of naturally occurring variations, it becomes possible to establish a framework for mapping and identifying modifier loci associated with the altered gene. As a result, we suggest executing a unique study by breeding SCIIMO mutant mice with various CC lines. This will produce F1 (SCIIMO+/− x CC) cells, which may have dramatically diverse susceptibilities to SCIIMO development (Figure 10). Our team successfully used a similar technique to map modifiers for the APC mutant gene, which is linked to the development of colorectal cancer [85].

To better comprehend the hereditary components that may be involved in the onset and development of class II malocclusion, a novel model for class II malocclusion should be developed while taking the diversity of the human population into consideration. For this objective, combining a CC model with an engineered class II malocclusion mouse model may reveal fresh information about the underlying genetics of the condition. To map and identify new modifier loci for the mutant/causative gene, a group of mice with a variety of naturally occurring genetic variations will be crossed with known class II malocclusion-associated mutants/genes. Parallel genetic platforms for in vitro/in vivo studies will be made possible—in particular, by creating F1 mouse models (SCIIMO mouse knockout gene crossed with CC mice (SCIIMO^ko^ X CC)), which will take advantage of the great genetic variety seen in the CC population, systematically revealing the essential characteristics in clinical findings. Class II malocclusion is connected with alterations in cell functions and molecules.

The system genetics analysis, which helps identify the genes, signaling pathways, and networks that underline common illnesses, has provided the first comprehensive evaluation of the molecular basis of complex traits. Data on the cellular, molecular, and clinical features are then combined to evaluate the relationships between distinct class II malocclusion phenotypes. When the SNP genotype information from each CC lineage is merged, the regulatory genomic regions are implicated in the phenotypic variations in both in vitro and in vivo monitored variables. Finding the genes associated with human class II malocclusion susceptibility may be possible by combining data with subsequent investigations on the relationships of candidate genes in humans. A better comprehension of how the interactions of various genetic alterations affect class II malocclusion initiation and severity may result from the possibility of parallel in vitro/in vivo screening in this experimental design, from the advancement of high-throughput assessment tools, and from computational approaches. To determine the likelihood of class II malocclusion and identify possible therapeutic targets, gene–gene interactions and/or gene–environment networks that have been verified may be implemented in human systems. By understanding the processes of the genetic loci (QTL and genes) discovered in the genome-wide association study (GWAS) that affect the vulnerability to class II malocclusion illnesses, system genetics will probably be able to recognize the pathophysiology of the disorder, as well as its severity.

**Figure 11:**
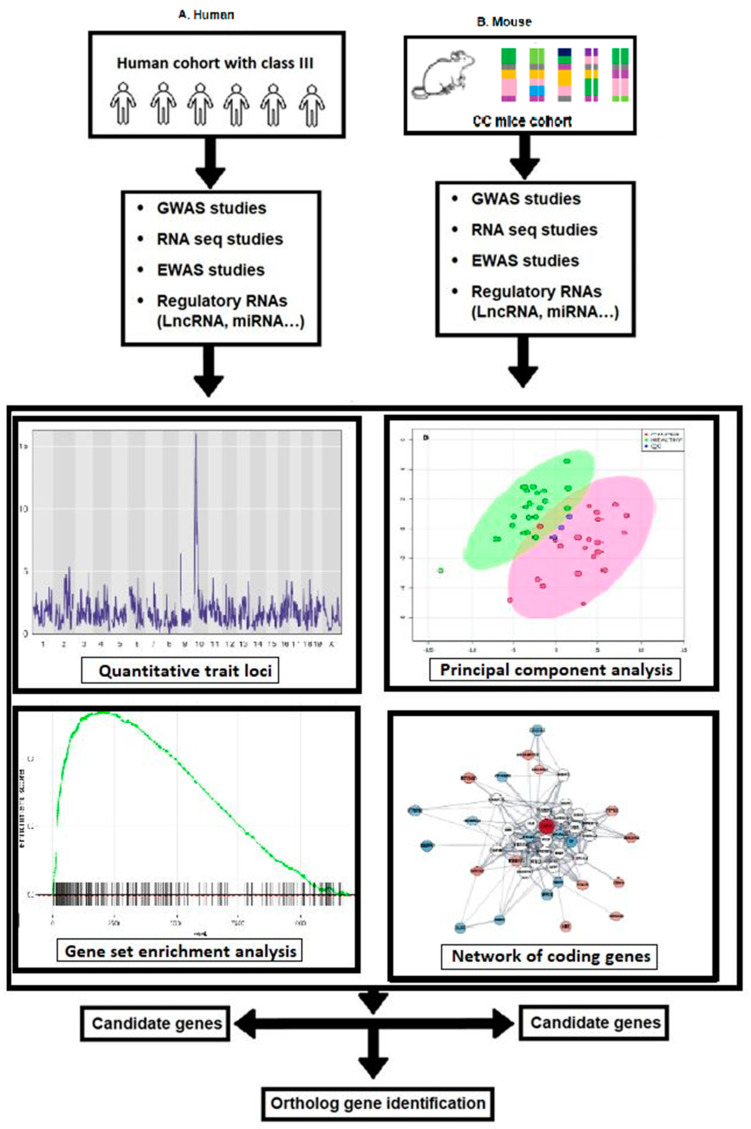
Workflow for generating system genetic datasets of cellular, molecular, and clinical trait data combined to analyze correlations between malocclusion and class II phenotypes. By integrating SNP genotype data, RNA expression, the regulatory genomic regions implicated in phenotypic variations of monitored traits, can be identified using QTL mapping in CC mouse models and humans. Combining these data with subsequent candidate gene association studies in humans has the potential to identify susceptibility genes associated with the development of class II malocclusion in humans.

Numerous studies are being conducted on regulatory RNAs at the molecular level right now, including studies of gene expressions, DNA methylation, short and microRNAs, and long noncoding RNA profiles, concerning numerous illnesses. However, to the best of our knowledge, very little research is known about the role these molecules play in skeletal class II malocclusion. Here, we propose that these regulatory RNAs will be crucial to investigate in this condition and will help us better understand the underlying molecular causes of this illness. A workflow diagram for the generation of system genetic datasets of cellular, molecular, and clinical trait data combined to analyze various correlations between malocclusion and class II phenotypes and the integration of human and mouse approaches, along with their identification, screening, and exclusion methods, is represented in Figure 11.

Finally, understanding how the genetic loci (QTL and genes) found in genome-wide association studies (GWAS) contributes to SCIIMO phenotype susceptibility and system genetics will likely allow a better understanding of both the biology and the disease.

## 5. Conclusions

When a therapist is informed of a class II malocclusion, they can better concentrate on their therapy planning and delivery. Developing a robust interincisal angle and excellent interdigitation of the occlusion is essential for achieving and maintaining a stable result. Stability improvement depends on long-term retention.

Using only methods intended to pinpoint the primary impacts of specific alleles in humans, it is thought that the genetic component of vulnerability to class II malocclusion cannot be fully understood. The complexity and heterogeneity of human class II malocclusion must therefore be studied, which necessitates the creation of new class II malocclusion mice models as a helpful model platform and resource. A possible starting point is a suggestion to perform a system genetics analysis utilizing CC GRP mice to see how genetic variations affect the signaling networks for class II malocclusion and phenotypic variety, along with performing a GWAS approach on a large subject population consisting of a well-characterized, multiethnic sample, including individuals with European, Middle East, Hispanic/Latino, admixed African, and Asian ancestry, will increase the power of the analysis and the opportunity to comprehensively identify genetic causes of skeletal class II malocclusion development in humans. It is strongly believed that applying the suggested parallel approaches to mice and humans will identify the genetic bases of SCIIMO development and subsequently offer novel approaches for the early detection and prevention strategies of this phenotype.

## Figures and Tables

**Table 1:** Presents a comprehensive overview, based on our current understanding, of the mapped QTL and their corresponding positions, as well as the range of significance, for the size (mean of the two sides) of the mandible characters (M), shape (SH), centroid size (C), and principal components (PCs) of the facial shape. The positions are indicated as map distances from both the nearest proximal marker and the centromere, determined through a QTL analysis conducted on mice.

Chr	CI (cM)	Reference
1	38–62	[72]
	78–120	[72]
	12–38	[73]
	49–65	[73]
	52–53	[74]
2	—	[72]
	—	[72]
	21–40	[73]
	62–70	[73]
3	37–53	[72]
	27–61	[72]
	14–24	[73]
	69–75	[73]
4	28–46	[72]
	18–42	[72]
	40–60	[73]
5	37–105	[72]
	26–62	[72]
	29–70	[73]
6	4–16	[72]
	74–98	[72]
	70–100	[72]
	3–19	[73]
	99–100.2	[74]
	3.5–6	[74]
7	13–65	[72]
	61–77	[72]
	1–85	[72]
	37–75	[74]
	58–66	[74]
8	16–36	[73]
9	16–38	[72]
	54–88	[72]
	41–65	[73]
10	9–41	[72]
	65–75	[72]
	63–87	[72]
	55–83	[72]
	16–28	[73]
	31–57	[73]
11	13–31	[72]
	63–97	[72]
	17–55	[72]
	67–109	[72]
	51–109	[72]
	4–47	[73]
12	21–33	[72]
	29–70	[72]
	43–63	[72]
	32–49	[73]
	8.5–17	[74]
13	79–97	[72]
	7–45	[72]
	37–59	[73]
14	48–64	[72]
	24–64	[72]
	5–27	[73]
	38–48	[73]
	22–24	[74]
15	29–53	[72]
	53–85	[72]
	41–67	[72]
	11–55	[72]
	27–43	[73]
16	14–44	[72]
	15–33	[73]
17	11–19	[72]
	29–45	[73]
	45–48	[74]
18	61–91	[72]
	25–73	[73]
19	15–61	[72]

## Data Availability

Not applicable.

## Abbreviations

QTL	Quantitative Trait Loci
RCTs	Research Clinical Trials
GWAS	Genome-Wide Associations
EWAS	Epigenetics-Wide Associations
FFA	Fixed Functional Appliances
SNPs	Single-Nucleotide Polymorphisms
*NOG* gene	noggin gene
RI	Recombinant Inbreed
GRP	Genetic Reference Population
CI	Confidence Interval
